# Autotrophic and mixotrophic metabolism of an anammox bacterium revealed by in vivo ^13^C and ^2^H metabolic network mapping

**DOI:** 10.1038/s41396-020-00805-w

**Published:** 2020-10-20

**Authors:** Christopher E. Lawson, Guylaine H. L. Nuijten, Rob M. de Graaf, Tyler B. Jacobson, Martin Pabst, David. M. Stevenson, Mike S. M. Jetten, Daniel R. Noguera, Katherine D. McMahon, Daniel Amador-Noguez, Sebastian Lücker

**Affiliations:** 1 Department of Civil and Environmental Engineering, University of Wisconsin-Madison, Madison, WI USA; 2 Department of Microbiology, Institute for Water and Wetland Research, Radboud University, Nijmegen, the Netherlands; 3 Department of Bacteriology, University of Wisconsin-Madison, Madison, WI USA; 4 DOE Great Lakes Bioenergy Research Center, University of Wisconsin-Madison, Madison, WI USA; 5 Department of Biotechnology, Delft University of Technology, Delft, The Netherlands

**Keywords:** Environmental microbiology, Metabolism

## Abstract

Anaerobic ammonium-oxidizing (anammox) bacteria mediate a key step in the biogeochemical nitrogen cycle and have been applied worldwide for the energy-efficient removal of nitrogen from wastewater. However, outside their core energy metabolism, little is known about the metabolic networks driving anammox bacterial anabolism and use of different carbon and energy substrates beyond genome-based predictions. Here, we experimentally resolved the central carbon metabolism of the anammox bacterium *Candidatus* ‘Kuenenia stuttgartiensis’ using time-series ^13^C and ^2^H isotope tracing, metabolomics, and isotopically nonstationary metabolic flux analysis. Our findings confirm predicted metabolic pathways used for CO_2_ fixation, central metabolism, and amino acid biosynthesis in *K. stuttgartiensis*, and reveal several instances where genomic predictions are not supported by in vivo metabolic fluxes. This includes the use of the oxidative branch of an incomplete tricarboxylic acid cycle for alpha-ketoglutarate biosynthesis, despite the genome not having an annotated citrate synthase. We also demonstrate that *K. stuttgartiensis* is able to directly assimilate extracellular formate via the Wood–Ljungdahl pathway instead of oxidizing it completely to CO_2_ followed by reassimilation. In contrast, our data suggest that *K. stuttgartiensis* is not capable of using acetate as a carbon or energy source in situ and that acetate oxidation occurred via the metabolic activity of a low-abundance microorganism in the bioreactor’s side population. Together, these findings provide a foundation for understanding the carbon metabolism of anammox bacteria at a systems-level and will inform future studies aimed at elucidating factors governing their function and niche differentiation in natural and engineered ecosystems.

## Introduction

Anaerobic ammonium oxidation (anammox) is a key step in the biogeochemical nitrogen cycle and represents a novel treatment process for the sustainable removal of nitrogen from wastewater. The process is mediated by a deeply branching group of chemolithoautotrophic bacteria within the Planctomycetes, the Brocadiales, that couple the anaerobic oxidation of ammonium to nitrite reduction and dinitrogen gas formation [1–4]. The discovery of this unique metabolism and subsequent translation to full-scale applications represents one of the most rapid biotechnological advances in wastewater treatment [5–7]. However, the metabolic networks supporting anammox metabolism remain poorly understood, which limits the prediction of their function in natural and engineered ecosystems.

Metagenomic sequencing together with experimental studies have begun to unravel the metabolic potential of anammox bacteria [2, 3, 8]. A combination of molecular approaches has revealed the key enzymes and reactions involved in anammox catabolism, which include hydrazine (N_2_H_4_) and nitric oxide as volatile intermediates in the anammox bacterium *Candidatus* “Kuenenia stuttgartiensis” (hereafter, *K. stuttgartiensis*) [3, 9, 10]. These reactions are localized within a specialized intracellular organelle, the anammoxosome, which is believed to be dedicated to energy conservation [11, 12] and also contains membrane-bound respiratory complexes of *K. stuttgartiensis*’ electron transport chain, including complex I, ATP synthase, and an ferredoxin:NAD^+^ oxidoreductase (RNF) [13]. Experimental studies together with genomic evidence have also suggested that anammox bacteria are much more versatile than initially assumed, and can use alternative electron donors to ammonium, such as formate, acetate, and propionate for energy conservation with nitrite or nitrate as electron acceptors [2, 8, 14–17]. Intriguingly, it has been proposed that these organic substrates are fully oxidized to CO_2_ and not directly assimilated into cell biomass, suggesting that anammox bacteria adhere to their autotrophic lifestyle [4].

**Fig. 1:**
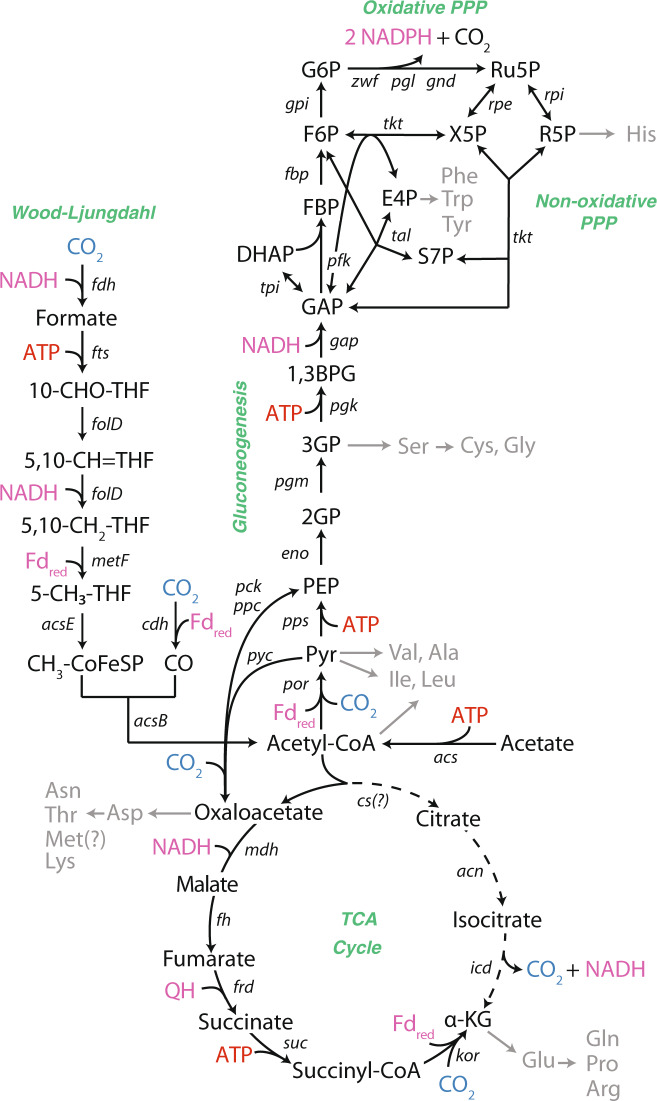
Predicted central metabolism of *K*. *stuttgartiensis* based on genome annotation. Black arrows represent reactions of the Wood–Ljungdahl pathway, gluconeogenesis, the TCA cycle, and the pentose phosphate pathway. Grey arrows indicate amino acid biosynthetic pathways. Pink metabolites represent reducing equivalents. Red metabolites indicate ATP equivalents. “*?”* indicates gene or pathway not identified in genome. A list of all reactions can be found in Supplementary Table 1. Confirmation of gene expression via proteomic analysis can be found in Supplementary Dataset 1.

Previous studies have shown that anammox bacteria use the Wood–Ljungdahl pathway to fix CO_2_ to acetyl-CoA based on cell carbon isotopic composition [18], genomic evidence [2, 19] and gene expression data [4]. Based on genome annotations, four additional carboxylation reactions are also predicted to incorporate CO_2_ during central carbon metabolism, catalyzed by pyruvate:ferredoxin oxidoreductase (PFOR), 2-oxoglutarate:ferredoxin oxidoreductase (OFOR), pyruvate carboxylase, and phosphoenolpyruvate carboxylase [2, 4] (Fig. 1; Supplementary Table 1). Products from these reactions are proposed to flow through the tricarboxylic acid (TCA) cycle, gluconeogenesis, and the pentose phosphate pathway to synthesize all biomass precursor metabolites [2, 4] (Fig. 1; Supplementary Table 1). Since the genome of *K. stuttgartiensis* lacks an annotated citrate synthase gene, it has been hypothesized that the TCA cycle operates in the reductive direction via OFOR to synthesize essential precursor metabolites, such as alpha-ketoglutarate [4] (Fig. 1; Supplementary Table 1). However, these genome-based predictions of *K. stuttgartiensis’* metabolic network remain to be experimentally investigated.

Here, we resolved the central carbon metabolism of a highly enriched planktonic *K. stuttgartiensis* cell culture using time-series ^13^C and ^2^H isotope tracing, metabolomics, and isotopically nonstationary metabolic flux analysis (INST-MFA). Our results map the in situ central carbon metabolic network of *K. stuttgartiensis* and show that several of the genome-based network predictions summarized above are not supported by the flux of metabolites experimentally observed. For instance, *K. stuttgartiensis* operates an oxidative TCA cycle despite its genome having no annotated citrate synthase. We also demonstrate that *K. stuttgartiensis* is able to directly assimilate formate via the Wood–Ljungdahl pathway instead of oxidizing it to CO_2_ prior to assimilation. Moreover, our data suggest that *K. stuttgartiensis* is not capable of using acetate as a carbon or energy source in situ and that acetate oxidation apparently occurred via the metabolic activity of a low-abundance microorganisms in the bioreactor’s microbial community. These findings contradict previous reports on the organic carbon metabolism of anammox bacteria and offer mechanistic insights on the versatility of carbon metabolism in *K. stuttgartiensis*.

## Results

### Mapping anammox autotrophic metabolism

To elucidate the central carbon metabolic network of the anammox bacterium *K. stuttgartiensis* under chemolithoautotrophic growth conditions, we first performed time-series isotopic tracer experiments with ^13^C-bicarbonate coupled to metabolomic analysis. Planktonic *K. stuttgartiensis* cells were cultivated under steady-state conditions in a 1 L continuous-flow membrane bioreactor using minimal media supplemented with 45 mM ammonium and nitrite (see “Materials and Methods”). The *K. stuttgartiensis* population was estimated to be ~97% of the total community biomass as determined by metaproteomic analysis (90% based on metagenomic analysis, Supplementary Dataset 2), which has been shown to be more representative of species biomass contributions than sequencing-based methods [20]. During steady-state cultivation, ^13^C-labelled bicarbonate was rapidly introduced into the bioreactor to a concentration of 30 mM (~65% ^13^C-dissolved inorganic carbon, DIC), which incorporated into *K. stuttgartiensis*’ metabolome over time. Samples were collected over a 3-h period to trace metabolic network structure based on rates of metabolite ^13^C-enrichment.

**Fig. 2:**
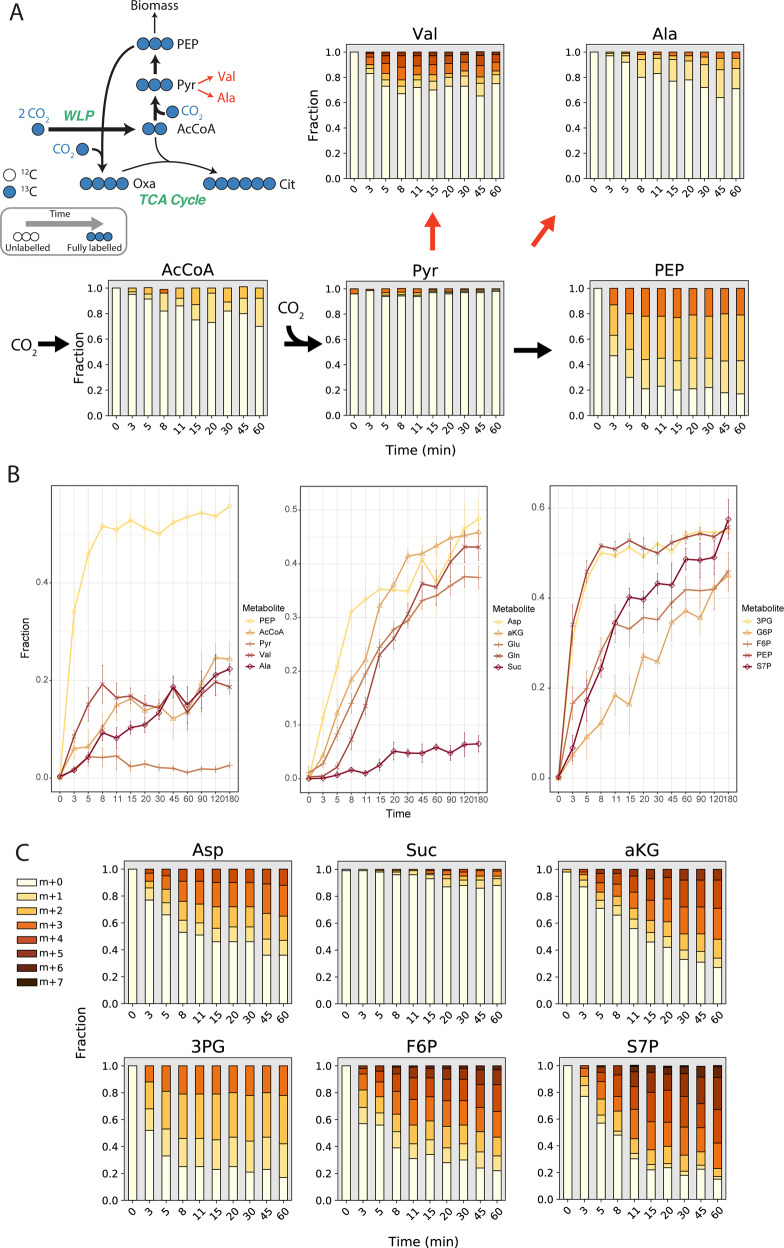
^13^C-enrichment of selected metabolites during ^13^C-bicarbonate dynamic tracing experiments. **a** Mass isotopomer distributions (MID) for selected metabolites illustrating the effects of potential substrate channelling or compartmentation of the Wood–Ljungdahl Pathway and PFOR. **b** ^13^C enrichment of metabolites associated with (left) initial CO_2_ fixation reactions (Wood–Ljungdahl Pathway, pyruvate:ferredoxin oxidoreductase) and metabolites downstream of pyruvate; (centre) TCA cycle metabolites; (right) gluconeogenesis and pentose phosphate pathway metabolites. **c** Selected mass isotopomer distributions for metabolites of the TCA cycle, gluconeogenesis, and the pentose phosphate pathway. All measured metabolite MIDs represent the average of 3 independent biological replicate experiments. Metabolite MIDs and standard errors can be found in Supplementary Dataset 3.

Based on the proposed carbon assimilation pathways for anammox bacteria [2, 4], we expected that CO_2_ fixation would largely occur through the Wood–Ljungdahl pathway and PFOR, resulting in fast labelling of acetyl-CoA and pyruvate, followed by phosphoenolpyruvate and other downstream metabolites. While complete labelling of phosphoenolpyruvate was observed almost immediately, ^13^C-enrichment of acetyl-CoA and pyruvate was slow during the 3-h experiment (Fig. 2a, b). One hypothesis for this observation is substrate channelling, where the product of one enzymatic reaction is directly passed to the next enzyme without opportunity to equilibrate within the cytoplasm [21]. An alternative explanation could be that different pools of acetyl-CoA and pyruvate exist through intracellular compartmentation, which would effectively dilute the measured overall ^13^C enrichment if one pool were inactive. Consistent with the latter, amino acids synthesized from pyruvate (i.e., valine and alanine) showed faster labelling and higher ^13^C-enrichment (Fig. 2a, b) than the pyruvate pool. This suggests that the Wood–Ljungdahl pathway and PFOR activities could occur in one compartment or specific cytoplasmic location [22] in *K. stuttgartiensis*, while other pools of acetyl-CoA and pyruvate that do not get labelled exist in another.

Following CO_2_ fixation, acetyl-CoA and pyruvate are expected to enter the TCA cycle and gluconeogenesis, respectively, to produce biomass precursors. Since *K. stuttgartiensis’* genome apparently does not encode a citrate synthase required to operate the oxidative TCA cycle, it is hypothesized that synthesis of key precursor metabolites, including succinyl-CoA and alpha-ketoglutarate, occurs via the reductive direction [4]. If this hypothesis is correct, we would expect to observe high ^13^C-labelling of oxaloacetate, succinate, and alpha-ketoglutarate. While fast labeling of aspartic acid, which was used as a proxy for oxaloacetate labelling, implied high activity of phosphoenolpyruvate (or pyruvate) carboxylase, ^13^C-labelling of succinate was much less and slower than the labelling of alpha-ketoglutarate (Fig. 2b, c). This suggested that the reductive TCA cycle was not operating in *K. stuttgartiensis*, and that alpha-ketoglutarate was rather synthesized via an unidentified citrate synthase and additional TCA cycle enzymes operating in the oxidative direction.

Biomass precursors are additionally predicted to be synthesized through gluconeogenesis and the pentose phosphate pathway in *K. stuttgartiensis* [2]. Consistent with this, we observed fast ^13^C-labeling of the gluconeogenic intermediates 3-phosphoglycerate, fructose 6-phosphate, and glucose 6-phosphate, as well as pentose phosphate pathway intermediates, such as sedoheptulose 7-phosphate and ribose 5-phosphate (Fig. 2b, c; Supplementary Dataset 3).

### ^13^C-formate tracing confirms formate assimilation pathways and oxidative branch of TCA cycle in *K. stuttgartiensis*

**Fig. 3:**
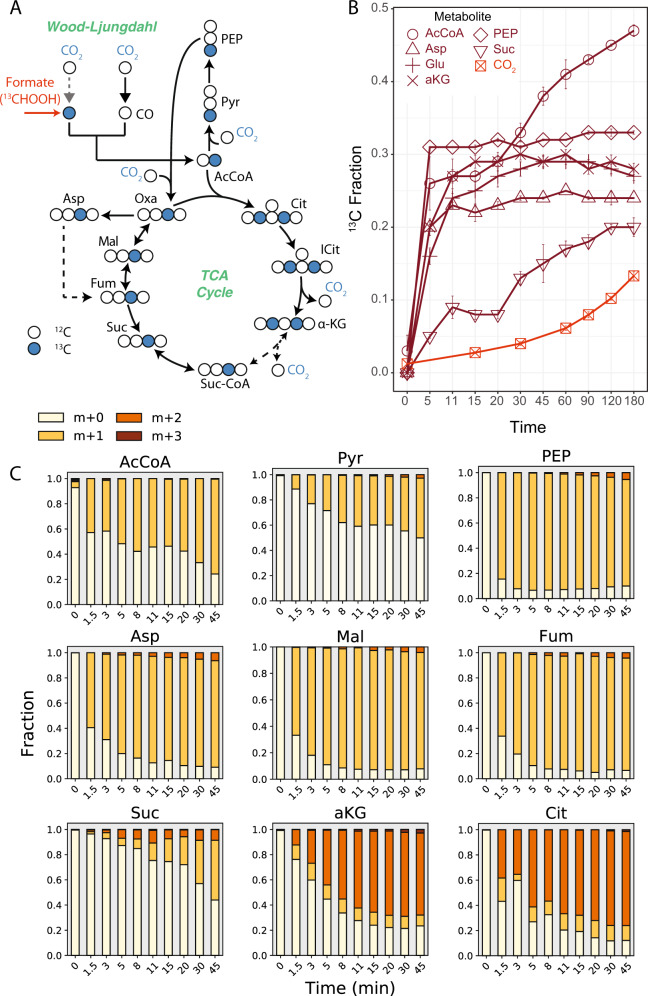
Elucidating TCA cycle of *K. stuttgartiensis* with ^13^C-formate. **a** Proposed labelling of TCA cycle metabolites with ^13^C-formate. **b** ^13^C-enrichment of selected metabolites during isotope tracer experiments with ^13^C-formate. **c** Time-series mass isotopomer distributions of selected TCA cycle metabolites during isotope tracer experiments with ^13^C-formate. All measured metabolite MIDs represent the average of 3 independent biological replicate experiments. Metabolite MIDs and standard errors can be found in Supplementary Dataset 3. Reaction carbon atom transitions are provided in Supplementary Dataset 4.

We further probed central carbon metabolism with ^13^C-formate. While it has been proposed that anammox bacteria fully oxidize organic substrates, such as formate, to CO_2_ [4], we hypothesized that formate could be assimilated by *K. stuttgartiensis* via the methyl branch of the Wood–Ljungdahl pathway. This would result in a positionally labelled acetyl-CoA pool that would provide additional information on metabolic network activity (Fig. 3a).

We tested this hypothesis by rapidly introducing ^13^C-formate into fresh continuous cultures of *K. stuttgartiensis* to a concentration of 50 mM, followed by metabolome sampling over 180 min (14 timepoints in total). Within 1.5 min of ^13^C-formate introduction, we observed steady-state labelling of several central metabolites, including phosphoenolpyruvate (Fig. 3b), consistent with direct assimilation of formate. In agreement with formate assimilation via the Wood–Ljungdahl pathway, only the M + 1 mass isotopomer of acetyl-CoA became enriched during the experiment (Fig. 3c). M + 1 mass isotopomers of phosphoenolpyruvate and aspartic acid (proxy for oxaloacetate) were also dominant (Fig. 3c), consistent with their synthesis from acetyl-CoA via the sequential reactions of PFOR, phosphoenolpyruvate synthase, and phosphoenolpyruvate **(**or pyruvate) carboxylase, respectively. Since only very minor fractions of the M + 2 mass isotopomers were detected in these metabolites (<3% over the initial 45 min), it can be concluded that intracellular ^13^C-CO_2_ concentrations remained low during the experiment. Consistent with this, measured ^13^C-DIC content in the liquid media produced from ^13^C-formate oxidation was low, increasing to only 5% over 45 min (Fig. 3b). This supports the inference that ^13^C-inorganic carbon incorporation into metabolites was insignificant compared to the rate of incorporation via ^13^C-formate. Similar to ^13^C-bicarbonate experiments, slower labelling of acetyl-CoA and pyruvate was observed during the ^13^C-formate tracer experiments (Fig. 3b, c) compared to downstream metabolites. This further supports the hypothesis that separate intracellular pools of these metabolites may exist in *K. stuttgartiensis*.

^13^C-formate labelling experiments also allowed us to analyze operation of the TCA cycle. If the TCA cycle was operating in *K. stuttgartiensis* only in the reductive direction, a single carbon in alpha-ketoglutarate would be labelled (from oxaloacetate), while two carbons would be labelled if alpha-ketoglutarate was produced oxidatively (from oxaloacetate and acetyl-CoA). Consistent with the latter route, mass isotopomer distributions for citrate and alpha-ketoglutarate consisted largely of M + 2 mass isotopomers (Fig. 3c). This clearly demonstrates that alpha-ketoglutarate was produced via oxidative TCA cycle reactions, and not via the reductive branch. On the contrary, malate, fumarate, and succinate pools were largely comprised of M + 1 mass isotopomers (Fig. 3c), which suggests that *K. stuttgartiensis* employs a bifurcated TCA cycle.

The labelling patterns of TCA cycle metabolites suggest that *K. stuttgartiensis* uses a novel or highly divergent enzyme for citrate synthesis. While no citrate synthase is annotated in the *K. stuttgartiensis* genome (NCBI ID: LT934425.1) [2, 23], several acyltransferase candidates exist, including genes annotated as (R)-citramalate synthase (KSMBR1_RS19040) believed to be involved in isoleucine biosynthesis [24] and redundant copies of 2-isopropylmalate synthase (KSMBR1_RS18315 and KSMBR1_RS10820). In particular, one of the 2-isopropylmalate synthase genes (KSMBR1_RS10820) is highly similar (55.1% identity) to the *Re*-citrate synthase identified in *Clostridium kluyveri* [25] and is located next to an ADP-forming succinate-CoA ligase of the TCA cycle. Therefore, we posit that this gene encodes a dedicated *Re*-citrate synthase that allows the oxidative TCA cycle to be operational in *K. stuttgartiensis*, a finding that calls for biochemical verification.

### Amino acid biosynthetic pathways

^13^C-formate tracer results were also used to confirm major amino acid biosynthetic pathways in *K. stuttgartiensis*, which were largely consistent with the genome-derived predictions. Our data support the synthesis of aspartate, asparagine, and threonine via canonical routes from oxaloacetate; the synthesis of glutamate, glutamine, proline, and arginine from alpha-ketogluturate; and the synthesis of serine from 3-phosphoglycerate (Supplementary Fig. 1). Furthermore, labelling patterns supported the production of alanine, valine, and leucine from pyruvate, and of the aromatic amino acids phenylalanine and tyrosine from erythrose 4-phosphate and phosphoenolpyruvate via the shikimate pathway (Supplementary Fig. 1). Interestingly, isoleucine biosynthesis was not supported by canonical routes from threonine, but rather via a recently described citramalate-dependent pathway from acetyl-CoA and pyruvate [26, 27] (Supplementary Fig. 2).

Despite the *K. stuttgartiensis* genome lacking an annotated pathway for methionine biosynthesis, methionine was labelled during both ^13^C-formate and ^13^C-bicarbonate experiments. Canonical precursors for methionine biosynthesis include aspartic acid and methyl-THF (from formate via the methyl-branch of Wood-L jungdahl pathway), thus if this pathway were operating, we would expect to see mainly M + 2 methionine. However, a considerable pool of M + 1 methionine was consistently observed in our experiments (Supplementary Fig. 1), suggesting that a potentially novel pathway to synthesize methionine is operating in *K. stuttgartiensis*, which remains to be elucidated.

### Isotopically non-stationary metabolic flux analysis of autotrophic growth

**Fig. 4:**
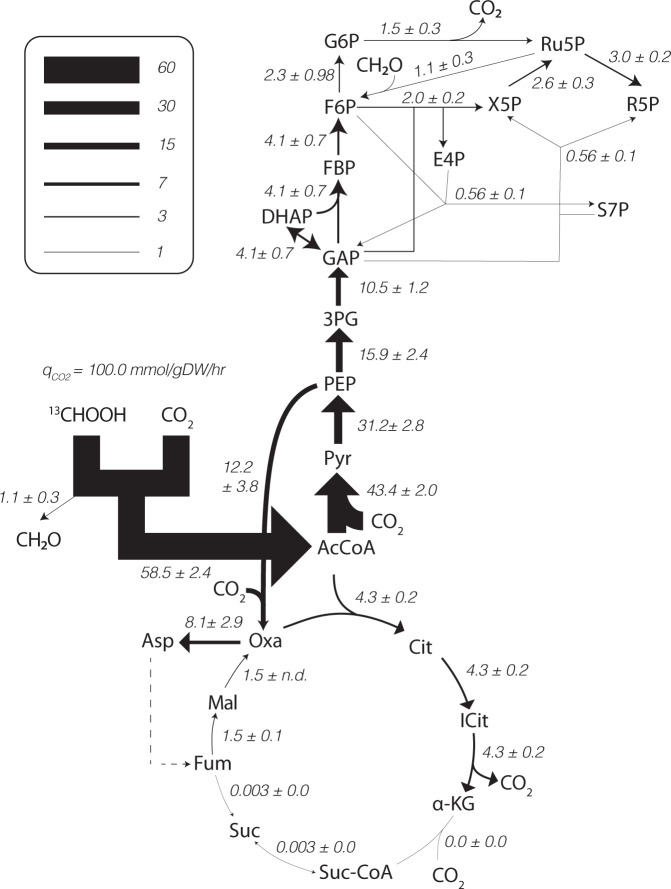
*K. stuttgartiensis* flux map generated by ^13^C INST-MFA. *K. stuttgartiensis* flux map under anaerobic, continuous flow, ammonium and nitrite medium conditions determined by fitting metabolites labelled with ^13^C-formate tracers to a single, statistically acceptable isotopomer network model. Flux values represent the net flux through a given reaction ± standard error defined at 95% confidence. All fluxes are normalized to a net CO_2_ uptake rate of q = 100 mmol-C (g DW)^−1^ h^−1^ (actual CO_2_ uptake rate was 0.186 mmol-C (g DW)^−1^ h^−1^). All isotopomer network model reactions are provided in Supplementary Dataset 4. INST-MFA solutions are provided in Supplementary Dataset 5.

To quantitatively examine *K. stuttgartiensis*’ central carbon metabolism and obtain intracellular flux measurements, we performed INST-MFA by fitting measured, time-resolved metabolite mass isotopomer distributions from ^13^C-formate tracer experiments to an isotopomer network model [28]. Timepoints for INST-MFA were selected within the first hour after ^13^C-formate introduction, where metabolism was assumed to be at quasi-steady state based on stable rates of anammox activity (Supplementary Fig. 3). This provided a quantitative systems-level flux map of *K. stuttgartiensis’* inferred central carbon metabolism (Fig. 4). Flux values were normalized to a net CO_2_ uptake rate that was estimated from the growth rate and cell carbon content: 0.0042 h^−1^ × 45 mmol-C (g DW)^−1^ = 0.186 mmol-C (g DW) ^−1^ h^−1^. The resulting flux map reproduces the high intracellular flux anticipated through the Wood–Ljungdahl pathway, PFOR, and phosphoenolpyruvate (or pyruvate) carboxylase, which are the main CO_2_ fixation reactions that we observed in *K. stuttgartiensis* (Fig. 4). INST-MFA also supported alpha-ketoglutarate production via the oxidative TCA cycle. Moreover, instead of running a bifurcated TCA cycle, the INST-MFA analysis predicts that the M + 1 isotopomers of fumarate, succinate, and malate were indirectly derived from aspartic acid as a result of histidine and arginine biosynthesis (Fig. 4). This suggests that the TCA cycle in *K. stuttgartiensis* operates incompletely, essentially functioning to produce the amino acid precursor alpha-ketoglutarate and recycle intermediates of amino acid biosynthesis. We also detect carbon flux through the oxidative pentose phosphate pathway (Fig. 3). This may be an important source of NAPDH generation in *K. stuttgartiensis*, especially since there is no annotated transhydrogenase in the genome (NCBI ID: LT934425.1).

INST-MFA also allowed us to further resolve metabolic pathways used for the biosynthesis of sugar phosphates in *K. stuttgartiensis*. Results from the ^13^C-formate tracer experiments revealed that a considerable fraction of fructose 6-phosphate was present as M + 1 mass isotopomers, which was unexpected as gluconeogenesis would result in largely M + 2 mass isotopomers (Supplementary Fig. 4). This suggested that alternative pathways may be operating to produce fructose 6-phosphate. The genome annotation of *K. stuttgartiensis* has genes encoding for hexulose 6-phosphate synthase and 6-phospho-3-hexuloisomerase (KSMBR1_RS05220 and KSMBR1_RS18790, respectively), which are key enzymes of the ribulose monophosphate (RuMP) pathway, a formaldehyde assimilation pathway in many methylotrophic bacteria [29]. Together, these reactions fix formaldehyde to fructose 6-phosphate via a hexulose 6-phosphate intermediate [29]. We hypothesize that these reactions, as well as an unidentified formaldehyde dehydrogenase, could explain the considerable M + 1 pentose and hexose phosphate isotopomers observed during ^13^C-formate labelling (Supplementary Fig. 5). Consistently, including these reactions in our INST-MFA improved the model’s fit by ~15% (SSR of 839.9 versus 988.7, 95% confidence interval), and the model predicted that they accounted for ~23% of the flux synthesizing fructose 6-phosphate (Fig. 4).

### ^13^C and ^2^H acetate tracing suggests *K. stuttgartiensis* does not utilize acetate as a substrate in situ

In addition to formate, we also examined the impact of acetate on *K. stuttgartiensis*’ metabolic network. While it has been proposed that anammox bacteria can oxidize acetate to CO_2_ [8, 30], the pathways used for acetate oxidation and whether or not acetate is assimilated into biomass has yet to be resolved. The oxidation of acetate to CO_2_ could be mediated by reversal of the Wood–Ljungdahl pathway in *K. stuttgartiensis*, as previously suggested for other anaerobic chemolithoautotrophic bacteria [31, 32], or via the oxidative TCA cycle. Alternatively, a different organism present at low abundance in the bioreactor’s microbial community may be responsible for acetate oxidation.

**Fig. 5:**
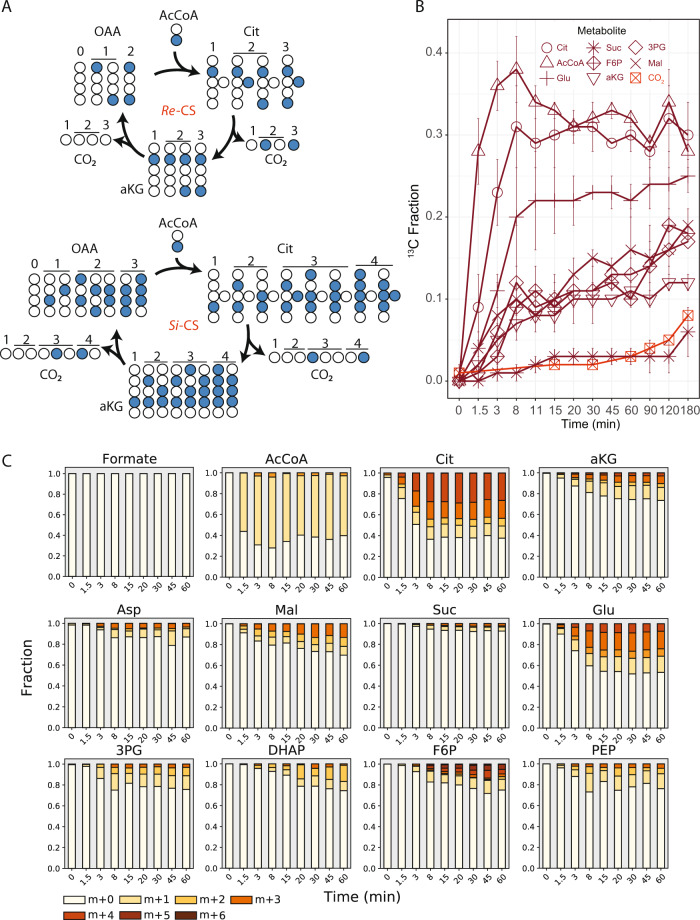
^13^C-acetate tracing reveals acetate is oxidized by TCA cycle and not reverse Wood–Ljungdahl pathway. **a** Expected dynamic labeling patterns of the TCA cycle based on either *Re*- or *Si*-citrate synthase from [2-^13^C]acetate tracing. Numbers above carbon molecules indicate how many rounds of the TCA cycle have been completed. For simplicity, only new isotopomers produced at each round of the TCA cycle are shown. **b** ^13^C-enrichment of selected metabolites during isotope tracer experiments with [2-^13^C]acetate (red). **c** Time-series mass isotopomer distributions of selected TCA cycle metabolites during isotope tracer experiments with ^13^C-acetate. All measured metabolite MIDs represent the average of 2 independent biological replicate experiments. Metabolite MIDs and standard errors can be found in Supplementary Dataset 3. Reaction carbon atom transitions are provided in Supplementary Dataset 4.

To elucidate metabolic pathways involved in acetate metabolism, we first rapidly introduced [2-^13^C]acetate into active continuous cultures of *K. stuttgartiensis* to a final concentration of 10 mM and sampled the metabolome over 180 min (12 timepoints). Within 1.5 min after [2-^13^C]acetate addition, we observed steady-state enrichment of the M + 1 mass isotopomer of acetyl-CoA, indicating its synthesis via CoA acetylation (Fig. 5), which appeared consistent with previous enzymatic studies confirming the activity of an AMP-forming acetyl-CoA synthetase in vitro (KSMBR1_RS14485) [33].

If acetyl-CoA was subsequently oxidized by the reverse Wood–Ljungdahl pathway, formate would become labeled, as it is a pathway intermediate. During our experiment, however, formate remained unlabeled (Fig. 5c), indicating that the reverse Wood–Ljungdahl pathway was not involved in acetyl-CoA oxidation to CO_2_. In contrast, ^13^C-labelling of TCA cycle and gluconeogenic metabolites occurred (Fig. 5c), although to a much lesser extent compared to results from ^13^C-formate tracing (Fig. 2). The observed labelling patterns for TCA cycle metabolites could be explained by multiple rounds of the oxidative TCA cycle employing a *Si*-citrate synthase, but not a *Re*-citrate synthase (Fig. 5a). In addition, the labelling patterns detected in gluconeogenic intermediates could be best explained by decarboxylation of labelled oxaloacetate followed by gluconeogenesis from phosphoenolpyruvate.

**Fig. 6:**
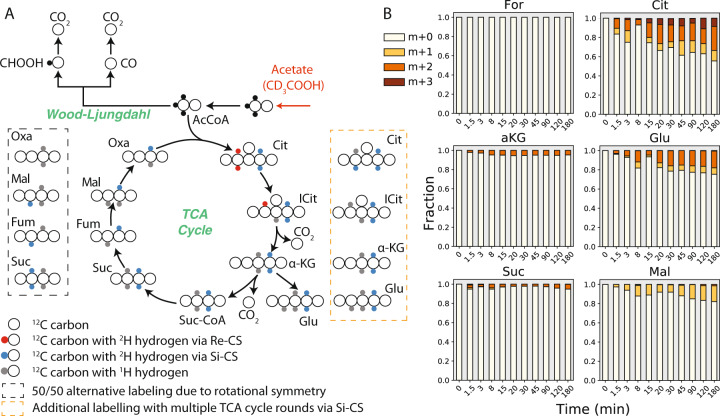
^2^H-acetate tracing suggests acetate oxidation by low abundance organism via *Si*-citrate synthase. **a** Proposed pathways and deuterium labelling for acetate oxidation via the reverse Wood–Ljungdahl pathway and TCA cycle. Black circles indicate carbons with heavy (^2^H) hydrogen, red circles indicate carbons with ^2^H hydrogen produced via *Re*-citrate synthase, blue circles indicate carbons with ^2^H hydrogen produced via *Si*-citrate synthase, grey circles indicate carbons with ^1^H (unlabelled) hydrogen. **b** Time-series mass isotopomer distributions of selected TCA cycle metabolites during isotope tracer experiments with sodium acetate-d3 (CD_3_COOH). All measured metabolite MIDs represent the average of 2 independent biological replicate experiments. Metabolite MIDs and standard errors can be found in Supplementary Dataset 3. Reaction hydrogen atom transitions are provided in Supplementary Dataset 4.

The observation of *Si*-citrate synthase activity is in contrast to the finding of a *Re*-citrate synthase gene candidate in the *K. stuttgartiensis* genome, suggesting that acetate may have been oxidized by a low abundance microorganism in the bioreactors side population. To obtain additional information on TCA cycle activity, we performed deuterium (^2^H) tracing experiments with 10 mM sodium acetate-d3 (CD_3_COOH, ^2^H-acetate). Following ^2^H-acetate addition to the reactor, formate again remained unlabeled over the course of the experiment, confirming that the reverse Wood–Ljungdahl pathway was inactive. In support of acetate oxidation via the TCA cycle, ^2^H labelling of TCA cycle metabolites was observed over 180 min, albeit at a very slow rate (Fig. 6). Similar to the ^13^C-acetate tracing results, ^2^H-acetate labelling patterns of TCA cycle metabolites were also consistent with the activity of a *Si*-citrate synthase, based on the presence of M + 2 succinate and alpha-ketoglutarate, and M + 1 malate (Fig. 6). This, together with the lack of label incorporation into the *K. stuttgartiensis* proteome (see below), suggests that acetate was oxidized by an unidentified microorganism in the bioreactor’s side population and not by *K. stuttgartiensis*. However, this requires further experimental investigation.

### ^13^C protein stable isotope probing confirms substrate uptake by *K. stuttgartiensis*

To confirm uptake of labelled substrates into the biomass of *K. stuttgartiensis* cells, we performed shotgun proteomics on peptides extracted from bioreactor biomass collected during the ^13^C-labelling experiments. Metaproteomic analysis of samples collected 0 and 72 h after label addition confirmed that ^13^C-bicarbonate was incorporated into the *K. stuttgartiensis* proteome, increasing at a median relative isotope abundance of ~50%, consistent with the ^13^C-DIC content of the liquid media (Supplementary Fig. 6). Incorporation of label from ^13^C-formate and [2-^13^C]acetate into the *K. stuttgartiensis* proteome was also detected after 72 h at median relative isotope abundances of ~30% and ~10%, respectively (Supplementary Fig. 6). These values are consistent with the use of the Wood–Ljungdahl pathway for formate assimilation (Fig. 3), and with fixation of ^13^C-CO_2_ produced from acetate oxidation (Fig. 5), as the ^13^C-DIC in the liquid media during the latter experiment held at ~10% between 5 and 72 h. Compared to the expected value of ~50% if [2-^13^C]acetate were assimilated, this low relative isotope abundance in the *K. stuttgartiensis* proteome thus lends further support to the interpretation that acetate was utilized by an unidentified microorganism in the bioreactor’s side population.

## Discussion

Elucidating the in vivo metabolic network of *K. stuttgartiensis* represents a major advance in understanding the central carbon metabolism of anammox bacteria. Our results provide the first measurements of metabolic flux via INST-MFA in an anammox bacterium, revealing a systems-level flux map of pathways involved in CO_2_ fixation, central metabolism, and amino acid biosynthesis in *K. stuttgartiensis*. We discovered that *K. stuttgartiensis* operates the oxidative branch of the TCA cycle likely mediated by *Re*-citrate synthase. This pathway operates incompletely in *K. stuttgartiensis* to synthesize alpha-ketoglutarate, similar to other anaerobic bacteria [34], and avoids the energetically costly use of reduced ferredoxin in the reductive TCA cycle. Furthermore, the considerable flux measured through the oxidative pentose phosphate pathway highlights an important link between carbon and energy metabolism for generating reducing equivalents (i.e. NADPH) in anammox bacteria.

Our analysis validated the use of the Wood–Ljungdahl pathway, PFOR, and phosphoenolpyruvate/pyruvate carboxylase for CO_2_ fixation in *K. stuttgartiensis*. The study also suggested possible compartmentalization and/or metabolic channelling of these enzymes. This may enable faster pathway kinetics or limit competition between competing reactions, as has been shown for other pathways [35, 36]. While we cannot definitely rule out that some of these effects may be influenced by metabolite exchange with low abundance microorganisms present in the reactor or kinetic isotope effects [37, 38], our acceptable network model fit via ^13^C INST-MFA indicates that all reactions were accounted for considering dilution fluxes.

In addition to CO_2_ fixation, our analysis demonstrated that *K. stuttgartiensis* can also directly use formate as a carbon source via assimilation through the Wood–Ljungdahl pathway. As formate is a common fermentation end-product found in anaerobic environments [39, 40], it is likely that anammox bacteria assimilate formate when available to lower the amount of reducing equivalents needed for CO_2_ fixation. In addition, formate oxidation via one of the formate dehydrogenases encoded by *K. stuttartiensis* [4] would contribute to energy conservation and provide low-potential electrons for carbon fixation, reducing the necessity to invest energy in reverse electron transport of electrons derived from nitrite oxidation. While this may confer ecological advantages in environments where formate is present, further studies that investigate the long-term effects of formate on *K. stuttartiensis* growth and substrate competition with heterotrophic bacteria are required.

Aside from formate, acetate has also been reported to be an organic substrate used by anammox bacteria [4, 15, 16, 33]. However, the low relative isotope abundance of *K. stuttartiensis* proteins from ^13^C-acetate labelling, in combination with the *Si*-citrate synthase activity indicated by ^13^C- and ^2^H-acetate tracer experiments, implies that *K. stuttgartiensis* was unable to use acetate as an electron donor or carbon source in situ. Instead, we posit that the observed acetate oxidation activity occurred via a low-abundance heterotrophic organism present in the bioreactor’s side population. This highlights the need to further understand the metabolism of organisms that co-occur with anammox bacteria in natural and engineered ecosystems [39, 40], and to explore pathways used by other anammox species, such as *Brocadia* spp., which have also been reported to use acetate as a substrate in situ [16, 30, 41].

While further studies are required to unravel the complete versatility of anammox bacteria, our study provides a foundation for understanding their carbon metabolism at a systems level. We expect that further mapping of carbon metabolism across different anammox species and under different environmental conditions will reveal key features underlying their niche differentiation in natural and engineered ecosystems.

## Materials and methods

### Cultivation of K. stuttgartiensis cells

A high enrichment of planktonic *K. stuttgartiensis* cells was cultivated in a continuous flow membrane bioreactor (MBR; Applikon Biotechnology B.V., The Netherlands), with working volumes ranging from 0.8 to 1.5 L [42]. The enrichment culture was estimated to contain ~97% *K. stuttgartiensis* based on metaproteomic analysis (see Supplementary Dataset 2). A summary of the metagenomic sequencing and binning statistics, including bin annotation and relative abundance, can be found in Supplementary Dataset 2.

The culture was fed a mineral salts medium consisting of 1.5 g L^−1^ KHCO_3_, 0.025 g L^−1^ KH_2_PO_4_, 0.5 ml L^−1^ 1.2 M HCl, 0.15 g L^−1^ CaCl_2_·2H_2_O, 0.1 g L^−1^ MgSO_4_·7H_2_O, 6.25 mg L^−1^ FeSO_4_·7H_2_O and 25 ml L^−1^ trace elements [43]. The media was supplemented with 45 mM of both ammonium and nitrite. Cultures were maintained under steady-state conditions at an OD_600_ of 1.0–1.2 via continuous biomass removal (~10% per day) and the bioreactor was continuously sparged with Ar/CO_2_ (95%/5% v/v) at a rate of 10 ml/min to maintain anaerobic conditions. The reactor hydraulic and solids retention times were ~48 h and 10.9 days, respectively. The temperature and pH of the reactor were controlled at 33°C and 7.3 using a heat exchanger and 1 M KHCO_3_ buffer, respectively. The reactor was continuously stirred at 160 rpm. Nitrite concentrations were checked daily to ensure nitrite-limited conditions (Nitrite test strips MQuant^®^, Merck, Darmstadt, Germany).

### Isotope tracer experiments

Isotope tracing experiments with ^13^C- and ^2^H-labelled substrates ([^13^C]sodium bicarbonate, [^13^C]sodium formate, [2-^13^C]sodium acetate, and sodium acetate-d3 (CD_3_COOH); Cambridge Isotopes Laboratories, MA, USA) were performed separately on continuous cultures of *K. stuttgartiensis* cells harvested from the MBR system. ^13^C bicarbonate and ^13^C formate tracing experiments were performed in triplicate; ^13^C and ^2^H acetate tracing experiments were performed in duplicate. For each isotope tracing experiment, *K. stuttgartiensis* cells were transferred anaerobically to adjacent 1 L MBRs within 30 min and immediately operated under identical steady-state conditions to those stated above. This resulted in no disruption of anammox activity, as determined by the absence of nitrite accumulation. Following 24 h of steady-state operation, ^13^C-labelled substrates were rapidly introduced (within 1 min) while gas inflow and outflow were clamped off. For the [^13^C]sodium bicarbonate experiments an acid pump for rapid buffering with 0.5 M anaerobic HCl was installed to maintain a pH of 7.3. Initial reactor concentrations of ^13^C-bicarbonate, ^13^C-acetate, ^2^H-acetate, and ^13^C-formate were ~30 mM, 5 mM, 10 mM, and 50 mM respectively. Following ^13^C-label introduction, 4 ml samples were rapidly withdrawn from the reactor at timepoints 0, 1.5, 3, 5, 8, 11, 15, 20, 30, 45, 60, 90, 120, and 180 min. Samples were immediately filtered (Whatman® 0.45 µm nylon membrane filters WHA7404004) using a vacuum pump to remove the medium, and filters were placed face down in 1.5 ml of −80°C extraction solvent (40:40:20 acetonitrile:methanol:water) for cell quenching and metabolite extraction. Cells were washed off the filter by pipetting the solvent over the filter surface 25 times. Samples were then transferred into 1.5 ml reaction tubes and centrifuged (10,000 rpm, 4°C, 5 mins) and 1 ml of cell-free supernatant was collected and stored at −80°C for metabolomic analysis. The time 0 min sample corresponded to the period directly before ^13^C-label addition. The ratio of ^13^C/^12^C DIC remained constant during the course of the experiment as determined by gas chromatography coupled with mass spectrometry (GC-MS) analysis (See below). Nitrite concentrations were checked at each sampling timepoint to ensure nitrite-limited conditions (Nitrite test strips MQuant®, Merck, Darmstadt, Germany).

For anammox activity measurements (Supplementary Fig. 3), the reactor was operated as described above with the exception that nitrite in the media was replaced with [^15^N]sodium nitrite (Cambridge Isotopes Laboratories, MA, USA). This allowed for monitoring of anammox activity via the production of ^14^N^15^N in the reactor headspace gas in the presence of unlabelled ammonium, based on the stoichiometry of anammox [8]. Production of ^14^N^15^N was also quantified via GC-MS.

### Metabolomic analysis

Samples were analysed using a high-performance HPLC–MS system consisting of a Vanquish^TM^ UHPLC system (Thermo Scientific) coupled by electrospray ionization (ESI; negative polarity) to a hybrid quadrupole high-resolution mass spectrometer (Q Exactive Orbitrap, Thermo Scientific) operated in full scan mode for detection of targeted compounds based on their accurate masses. Properties of Full MS–SIM included a resolution of 140,000, AGC target of 1E6, maximum IT of 40 ms and scan range from 70 to 1,000 m/z. LC separation was achieved using an ACQUITY UPLC BEH C18 column (2.1 × 100 mm column, 1.7 μm particle size; part no. 186002352; serial no. 02623521115711, Waters). Solvent A was 97:3 water:methanol with 10 mM tributylamine (TBA) adjusted to pH 8.1–8.2 with 9 mM acetic acid. Solvent B was 100% methanol. Total run time was 25 min with the following gradient: 0 min, 5% B; 2.5 min, 5% B; 5 min, 20% B; 7.5 min, 20% B; 13 min, 55% B; 15.5 min, 95% B; 18.5 min, 95% B; 19 min, 5% B; 25 min, 5% B. Flow rate was 200 μl min^–1^. The autosampler and column temperatures were 4°C and 25°C, respectively. Mass isotopomer distributions were corrected for natural abundance using the method of Su et al. [44] and ^13^C enrichment values were calculated using the formula $\left( {1/N} \right)\mathop {\sum}\nolimits_{i = 1}^N {Mi \times i}$, where *N* is the number of carbon atoms in the metabolite and *Mi* is the fractional abundance of the *i*^th^ mass isotopomer. Compounds were identified by retention time matching to pure standards and monoisotopic mass. Data were analyzed using the MAVEN software suite [45].

To improve separation and measurement sensitivity of specific central carbon metabolites and intracellular amino acids, samples were first derivatized with either aniline [46, 47] or benzyl chloroformate [48], respectively. For aniline derivatization, samples were resuspended in 50 µl HPLC-grade water, 5 µl aniline (6 M, pH 4.5), and 5 µl N-(3-dimethylaminopropyl)-N’-ethylcarbodiimide hydrochloride (200 mg/ml). After 2 h of incubation at room temperature, 1 µl of triethylamine was added to stop the reaction. For benzyl chloroformate derivatization, samples were resuspended in 10 µl HPLC-grade water, 40 µl methanol, 5 µl of triethylamine, and 1 µl benzyl chloroformate and incubated at room temperature for 30 min.

### GC-MS analysis of dissolved inorganic carbon isotopic fractions and reactor headspace gases

Isotopic fractions of DIC in the liquid media were measured based on a modified headspace method [49]. 3 ml of culture liquid were collected from the bioreactor with a syringe and directly filtered through a sterile 0.45 µm filter (Whatman, cellulose acetate) and 26 G needle into a 60 ml bottle containing 1 ml 6 M HCl and crimp sealed with a rubber stopper. Prior to adding the liquid sample, bottles and HCl were flushed with either 100% N_2_ or Ar gas to void the headspace of background CO_2_. Samples were equilibrated with the acid in the bottles for at least 1 hour at room temperature to drive all DIC into the gas phase. 50 µl of the bottles headspace was then injected with a gas tight syringe (Hamilton) into a gas chromatograph (Agilent 6890 equipped with 6 ft Porapak Q columns) at 80 °C with helium as a carrier gas at a flow rate of 24 ml min^−1^, coupled to a mass spectrometer (Agilent 5975 C MSD; Agilent, Santa Clara, CA) to determine the isotopic fractions of ^12^CO_2_ and ^13^CO_2_.

Reactor headspace gas samples were collected manually using a gas tight syringe and needle (Hamilton) through a rubber septum in the reactor headplate and directly injected into the GC-MS as described above.

### Isotopic non-stationary metabolic flux analysis

Intracellular metabolic fluxes were estimated from the measured metabolite isotope labelling dynamics via INST-MFA using the elementary metabolite unit method [28] implemented in the INCA software package v1.8 [50]. Metabolic fluxes and pool sizes were estimated by minimizing the lack-of-fit between measured and computationally simulated metabolite mass isotopomer distributions using least-squares regression. All metabolite mass isotopomer distribution measurements and model reactions used for flux determination are provided in Supplementary Datasets 3 and 4, respectively. The biomass equation was based on experimental measurements of the amino acid composition from *K. stuttgartiensis* biomass pellets for protein biosynthesis (Supplementary Table 2), while metabolite precursors for nucleic acid, lipid, and carbohydrate biosynthesis were assumed based on measurements from *E. coli* [51]. Dilution fluxes were added to the model for specific metabolites to account for inactive metabolite pools that did not participate in metabolism, but contributed to measured metabolite labelling patterns, similar to Ma et al. [52]. Chi-squared statistical tests were performed on resulting flux distributions to assess goodness-of-fit, and accurate 95% confidence intervals were computed for all estimated parameters by evaluating the sensitivity of the sum-of-squared residuals to parameter variations [53].

### Amino acid composition analysis

Cultures were centrifuged (10,000 rpm, 15 min, 4°C) to obtain cell pellets, which were subsequently freeze-dried prior to analysis. Total protein concentration was determined using the Pierce^TM^ BCA Protein Assay Kit (ThermoFisher Scientific) and amino acid composition was determined according to Carnicer et al. [54] using a Varian 920-LC high performance liquid chromatography amino acid analyzer.

### ^13^C protein stable isotope probing

Proteins were extracted from bioreactor cell pellets using glass bead beating (acid washed, 0.1 mm diameter) in a suspension containing B-PER reagent (Thermo Scientific, Germany) and TEAB buffer (50 mM TEAB, 1% (w/w) NaDOC at pH 8). Following DTT reduction and alkylation using iodo acetamide (IAA), protein extracts were subject to proteolytic digestion using trypsin. Resulting peptides were solid phase extraction-purified using an Oasis HLB 96 well plate (Waters, UK), according to the manufacturer protocols. The purified peptide fraction was analysed via a one-dimensional reverse phase separation (Acclaim PepMap RSLC RP C18, 50 µm x 150 mm, 2 µm, 100 A) coupled to a Q Exactive plus Orbitrap mass spectrometer (Thermo Scientific, Germany) operating in data-dependent acquisition mode (DDA, shotgun proteomics). The flow rate was maintained at 300 nL min^−1^ over a linear gradient from 5 to 30% solvent B over 90 min and finally to 75% B over 25 min. Solvent A was H_2_O containing 0.1% formic acid, and solvent B consisted of 80% acetonitrile in H_2_O and 0.1% formic acid. The Orbitrap was operated in DDA mode acquiring peptide signals from 350 to 1400 m/z, where the top 10 signals (with a charge between 2 and 7) were isolated at a window of 2.0 m/z and fragmented using a NCE of 30. The AGC target was set to 1E5, at a max IT of 54 ms and 17.5 K resolution. Protein identification and relative isotope abundances were determined from Tandem-MS data using PEAKS Studio X (BSI, Canada) and MetaProSIP (OpenMS, Univ Tuebingen/Berlin, Germany) [55] integrated into the KNIME 4.0.1 analytics platform (Zurich, Switzerland), respectively. All peptide spectra were matched against a protein database generated from predicted open reading frames from the total metagenomic assembly.

## Supplementary information

Supplementary Information

Supplementary Dataset 1

Supplementary Dataset 2

Supplementary Dataset 3

Supplementary Dataset 4

Supplementary Dataset 5
