# Supplementary material for: Autotrophic and mixotrophic metabolism of an anammox bacterium revealed by in vivo 13C and 2H metabolic network mapping
Source: ISME J. 2020 Oct 20;15(3):673–87. doi: 10.1038/s41396-020-00805-w (PMC8027424; doi:10.1038/s41396-020-00805-w)
Supplement: Supplementary file 1 — Supplementary Information [file 41396_2020_805_MOESM1_ESM.pdf]

SUPPLEMENTARY INFORMATION

Autotrophic and mixotrophic metabolism of an anammox bacterium revealed by *in vivo* ^{13}C and ^{2}H metabolic network mapping

Christopher E. Lawson[1,*], Guylaine H.L. Nuijten[2,#], Rob M. de Graaf [2,#], Tyler B. Jacobson[3], Martin Pabst[4], David. M. Stevenson[3], Mike S.M. Jetten[2], Daniel R. Noguera[1,5], Katherine D. McMahon[1,3], Daniel Amador-Noguez[3], Sebastian Lücker[2,*]

[1]Department of Civil and Environmental Engineering, University of Wisconsin-Madison, Madison, WI, USA
[2]Department of Microbiology, Institute for Water and Wetland Research, Radboud University, Nijmegen, the Netherlands
[3]Department of Bacteriology, University of Wisconsin-Madison, Madison, WI, USA
[4]Department of Biotechnology, Delft University of Technology, Delft, The Netherlands
[5]DOE Great Lakes Bioenergy Research Center, University of Wisconsin-Madison, Madison, WI, USA

[*]Corresponding authors: Christopher E. Lawson (c.e.lawson.87@gmail.com), Sebastian Lücker (s.luecker@science.ru.nl)

Supplementary Methods

Metagenomic sequencing and analysis

Metagenomic DNA was extracted from biomass samples collected from the anammox bioreactor at two independent timepoints (18/12/2019 and 19/08/2020) immediately prior to ^{13}C formate and ^{13}C acetate tracer experiments, respectively. DNA was extracted using the DNeasy PowerSoil Kit (Cat No./ID: 12888-100, Hilden, Germeny) and included bead beating with lysing matrix E (MP Biomedicals, California, USA). Metagenomic sequencing was performed on the Illumina HiSeq 2000 platform to generate 150bp paried-end reads. Paired-end Illumina reads were quality filtered using FastQC (http://www.bioinformatics.babraham.ac.uk/projects/fastqc/) and assembled using metaSpades version 3.10.1[1]. Reads were mapped to resulting contigs using bowtie2[2] to determine coverage. Automated binning with Metabat[3], Concoct[4], MaxBin2[5], BinSanity[6], and COCACOLA[7] using differential coverage was then performed and the best bins were selected using DAStool[8]. CheckM version 1.0.8 was used to determine bin completeness, redundancy, and taxonomy[9]. A summary of the metagenomic sequencing and binning statistics, including bin annotation and relative abundance, can be found in Supplementary Dataset 2. The relative abundance of the recovered genomes was calculated from the total number of DNA reads that mapped to the genome, divided by the genome length (read count/genome size).

Supplementary Dataset 1. Steady-state protein expression data for *K. stuttgartiensis* based on metaproteomic analysis.

Supplementary Dataset 2. Abundance of metagenome-assembled genomes (MAGs) based on metaproteomic analysis. (A) Summary of the MAGs recovered from the anammox membrane bioreactor investigated in this study. Replicate 1 was collected on 18/12/2019 immediately prior to a ^{13}C-formate tracer experiment; Replicate 2 was collected on 19/08/2020 immediately prior to a ^{13}C-acetate tracer experiment. The recovered *K. stuttgartiensis* genome (planctomycetaceae_1_das_tool) shared 99.6% genomic average nucleotide identity to the previously published *K. stuttgartiensis* genome (NCBI ID: LT934425.1) and is highlighted in bold text. (B) Quantification of MAG abundances based on the sum of peptide-spectrum matches (psm) from metaproteomic analysis.

Supplementary Dataset 3. Average metabolite mass isotopomer distributions and associated standard errors during ^{13}C-bicarbonate, ^{13}C-formate, [2-^{13}C]acetate, and sodium acetate-d3 tracer experiments. (Sheet 1A) average mass isotopomer distributions for selected metabolites during 13C-bicarbonate tracing; (Sheet 1B) mass isotopomer distributions standard error values for selected metabolites during 13C-bicarbonate tracing; (Sheet 2A) average mass isotopomer distributions for selected metabolites during 13C-formate tracing; (Sheet 2B) mass isotopomer distributions standard error values for selected metabolites during 13C-formate tracing; (Sheet 3A) average mass isotopomer distributions for selected metabolites during [2-^{13}C]acetate tracing; (Sheet 3B) mass isotopomer distributions standard error values for selected metabolites during [2-^{13}C]acetate tracing. (Sheet 4A) average mass isotopomer distributions for selected metabolites during sodium acetate-d3 tracing, (Sheet 4B) mass isotopomer distributions standard error values for selected metabolites during sodium acetate-d3 tracing.

Supplementary Dataset 4. *K. stuttgartiensis* isotopomer network model. Sheet 1 (Model): Letters within brackets indicate carbon atom transitions of each metabolite for a given reaction. Sheet 2 (2H_mapping): Capital letters within brackets indicate carbon transitions of each metabolite for a given reaction, lowercase letters indicate hydrogen transitions.

Supplementary Dataset 5. INST-MFA model results. Metabolite MIDs used for model fitting were Pro, Asn, Ala, Thr, aKG, Ser, Suc, Asp, Glu, R5P, PEP, Cit, Mal, Ru5P, Fum, F6P, Pyr, G6P, Val, CO_2, and Gln at timepoints 0, 1.5, 3, 5, 8, 11, 15, 20, 30, and 45 minutes. All metabolite MIDs can be found in Supplementary Dataset 3.

Reference

1. Nurk, S., Meleshko, D., Korobeynikov, A. & Pevzner, P. A. metaSPAdes : a new versatile metagenomic assembler. *Genome Res.* **27**, 824–834 (2017).
2. Langmead, B. & Salzberg, S. L. Fast gapped-read alignment with Bowtie 2. *Nat. Methods* **9**, 357–359 (2012).
3. Kang, D. D., Froula, J., Egan, R. & Wang, Z. MetaBAT, an efficient tool for accurately reconstructing single genomes from complex microbial communities. *PeerJ* **3**, e1165

(2015).

4. Alneberg, J. *et al.* Binning metagenomic contigs by coverage and composition. *Nat. Methods* **11**, 1144–1146 (2014).
5. Wu, Y.-W., Tang, Y.-H., Tringe, S. G., Simmons, B. a & Singer, S. W. MaxBin: an automated binning method to recover individual genomes from metagenomes using an expectation-maximization algorithm. *Microbiome* **2**, 26 (2014).
6. Graham, E. D., Heidelberg, J. F. & Tully, B. J. BinSanity: unsupervised clustering of environmental microbial assemblies using coverage and affinity propagation. *PeerJ* **5**, e3035 (2017).
7. Lu, Y. Y., Chen, T., Fuhrman, J. A. & Sun, F. COCACOLA: binning metagenomic contigs using sequence COmposition, read CoverAge, CO-alignment and paired-end read LinkAge. *Bioinformatics* **33**, 791–798 (2017).
8. Sieber, C. M. K. *et al.* Recovery of genomes from metagenomes via a dereplication, aggregation and scoring strategy. *Nat. Microbiol.* **3**, 836–843 (2018).
9. Parks, D. H., Imelfort, M., Skennerton, C. T., Hugenholtz, P. & Tyson, G. W. CheckM: assessing the quality of microbial genomes recovered from isolates, single cells, and metagenomes. *Genome Res.* **25**, 1043–55 (2015).

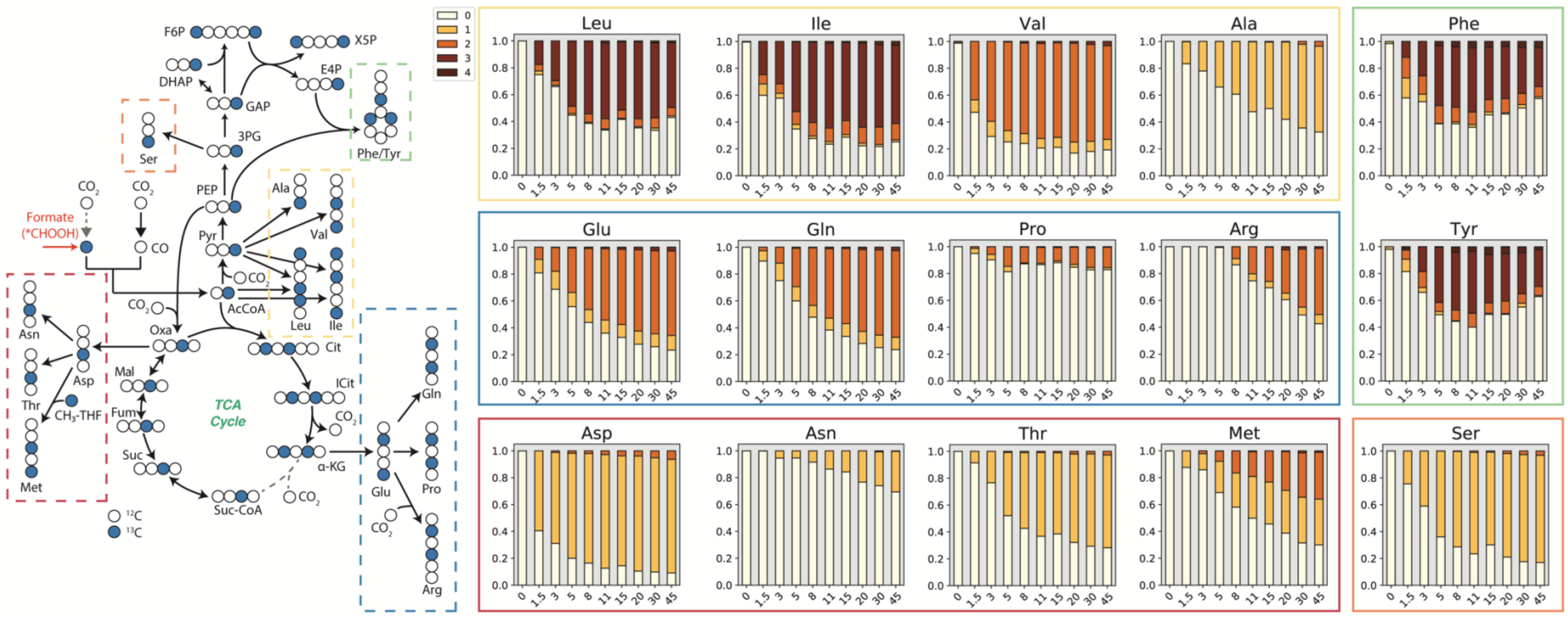

Supplementary Figure 1. Confirmation of amino acid biosynthetic pathways in *K. stuttgartiensis*. (Left) Expected metabolite labeling patterns from ^{13}C-formate. (Right) Mass isotopomer distributions for measured intracellular amino acids. All measured metabolite MIDs represent the average of 3 independent biological replicates experiments. Metabolite MIDs and standard errors can be found in Supplementary Dataset 3.

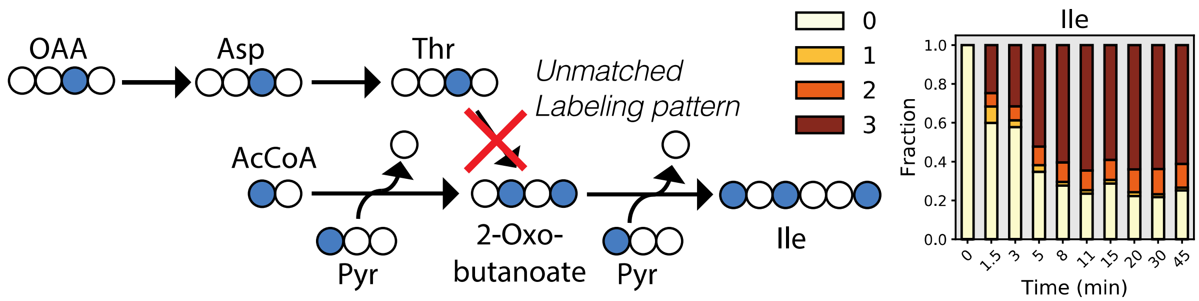

Supplementary Figure 2. Biosynthesis of isoleucine via citramalate-dependent pathway from acetyl-CoA and pyruvate. Blue circles indicate ^{13}C-labelled carbons from ^{13}C-formate.

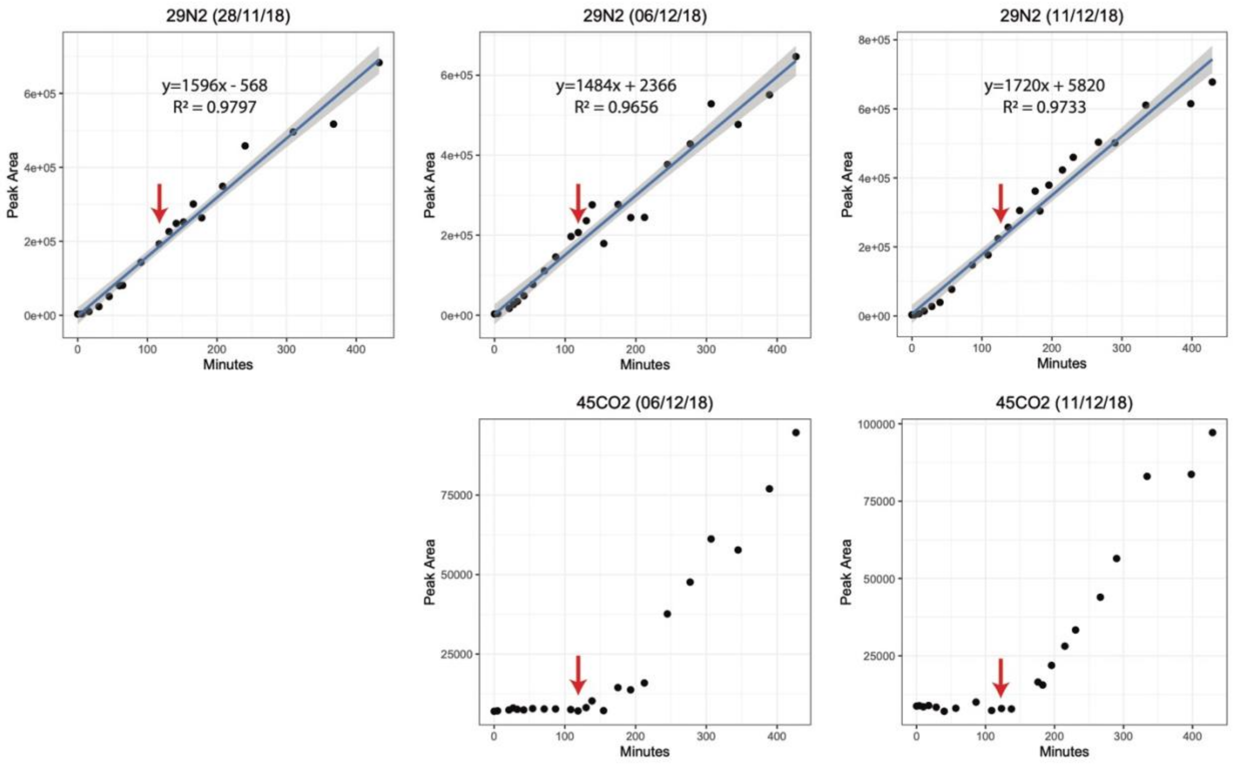

Supplementary Figure 3. (Top) Anammox activity measurements based on the production of $^{14}N^{15}N$ ($^{29}N_2$) in the reactor headspace with ^{15}N-nitrite and unlabelled (^{14}N) ammonium in the liquid media. Anammox reaction stoichiometry: $^{14}NH_4^+ + ^{15}NO_2^- \rightarrow ^{29}N_2$. (Bottom) Production of ^{13}C-labelled CO_2 ($^{45}CO_2$) from ^{13}C-formate oxidation. Red arrows indicate timepoint of ^{13}C-formate addition to reactor. Text in brackets indicates date of experiment (dd/mm/yy).

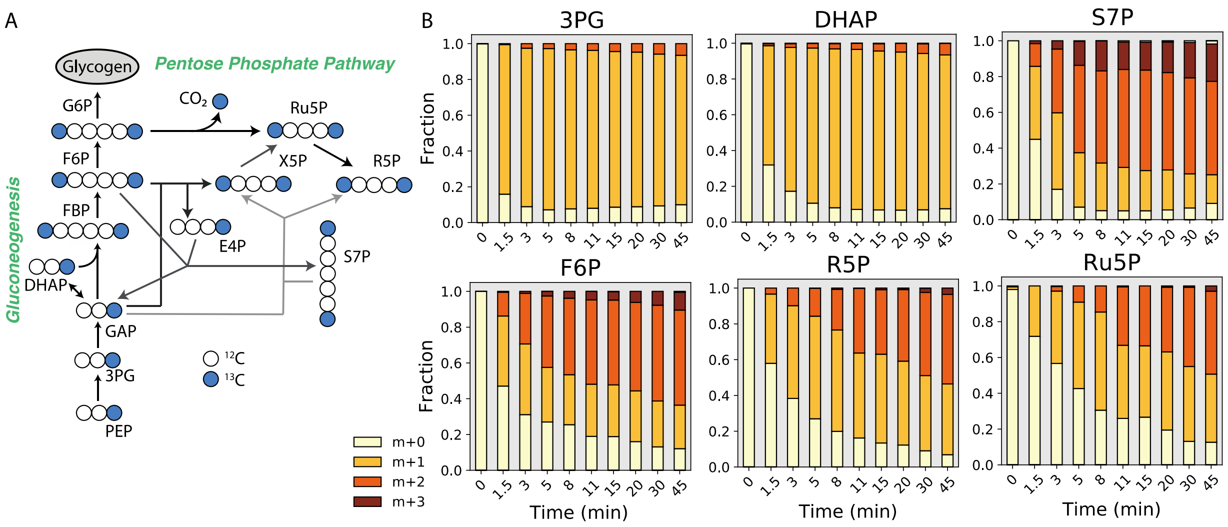

Supplementary Figure 4. Operation of gluconeogenesis and pentose phosphate pathway in *K. stuttgartiensis* revealed by ^{13}C-formate dynamic labelling experiments. (A) Proposed atom mapping of gluconeogenesis and pentose phosphate pathway from ^{13}C-formate labelled phosphoenolpyruvate at steady-state. (B) Time-series mass isotopomer distributions of selected gluconeogenesis and pentose phosphate pathway metabolites during dynamic isotope tracer experiments with ^{13}C-formate. All measured metabolite MIDs represent the average of 3 independent biological replicate experiments. Metabolite MIDs and standard errors can be found in Supplementary Dataset 3.

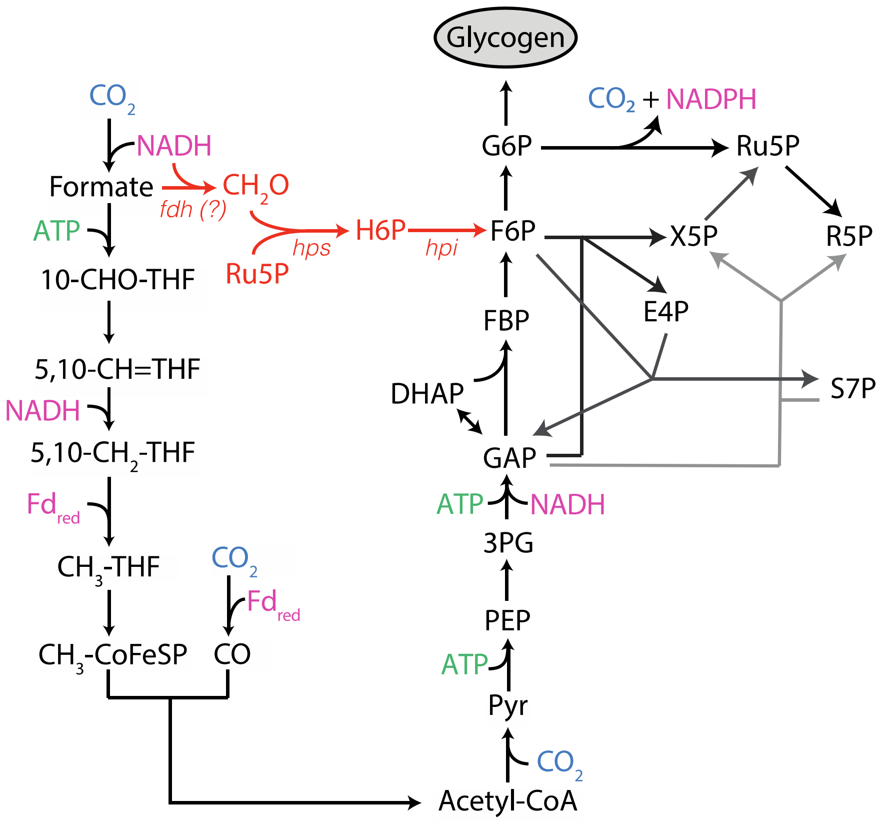

Supplementary Figure 5. Proposed synthesis of sugar phosphates from gluconeogenesis and the RuMP cycle in *K. stuttgartiensis*. RuMP cycle reactions that synthesize fructose 6-phosphate (F6P) from formaldehyde (CH_2O) and ribulose 5-phosphate (Ru5P) are shown in orange. Production of CH_2O from formate via an unknown formaldehyde dehydrogenase is also show in orange. Reducing equivalents shown in pink; ATP shown in green; CO_2 shown in blue. *fdh*: formaldehyde dehydrogenase; *hps*: hexulose 6-phosphate synthase; *hpi*: hexulose 6-phosphate isomerase; *?*: gene not identified in genome.

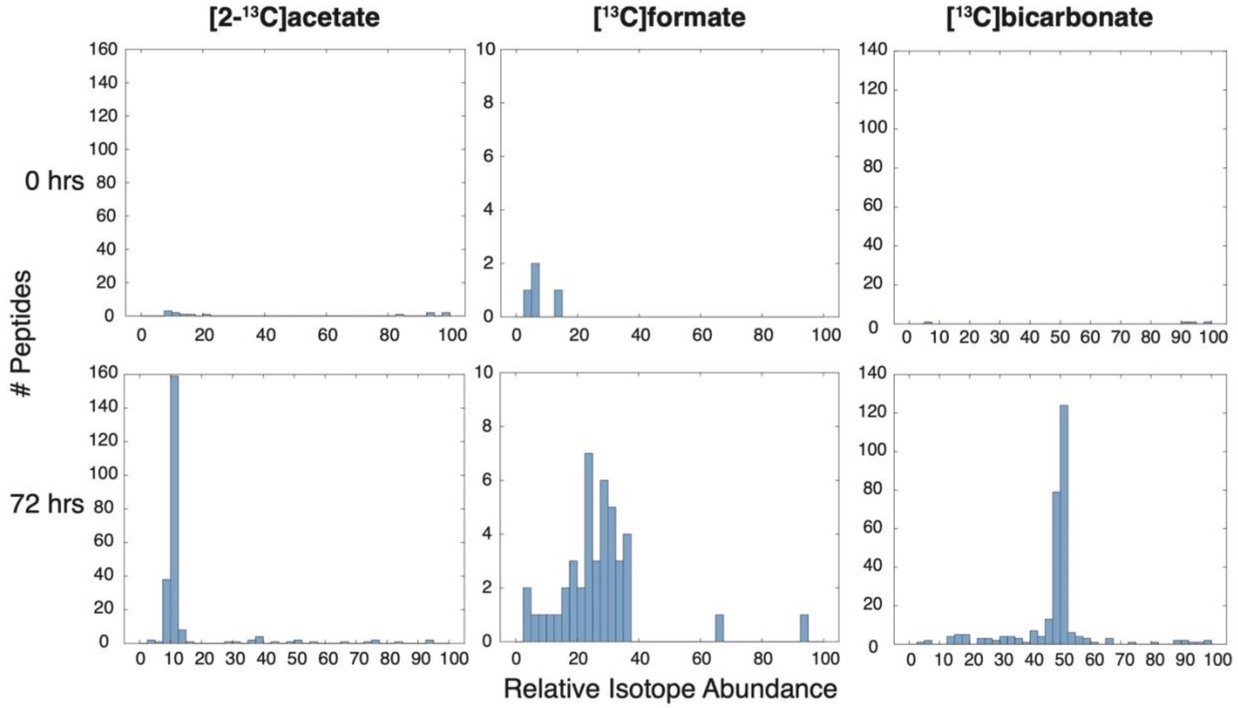

Supplementary Figure 6. Confirmation of ^{13}C-labelled substrate incorporation into the proteome of *K. stuttgartiensis*. Distribution of relative isotope abundances for identified peptides assigned to the *K. stuttgartiensis* proteome during [^{13}C]bicarbonate, [^{13}C]formate, [2-^{13}C]acetate tracer experiments after 0 and 72 hours. Fraction of dissolved $^{13}C\text{-}CO_2$ in the liquid media after 72 hours following [^{13}C]bicarbonate, [^{13}C]formate, and [2-^{13}C]acetate addition was 61% ± 5.7, 41% ± 5%, and 10% ± 0.9%, respectively.

gene	locus_tag	name
TCA cycle		
acnA	KSMBR1_2725	aconitase 1 (aconitate hydratase 1; citrate hydro-lyase 1)
icdA	KSMBR1_3471	similatr to isocitrate dehydrogenase
korC	KSMBR1_1008	oxoacid:ferredoxin oxidoreductase gamma chain
sucCD	KSMBR1_2158-2159	succinyl CoA-synthetase
sdhABC	KSMBR1_2357-2359	succinate dehydrogenase
frdB	KSMBR1_2005	succinate dehydrogenase/fumarate reductase (chain B) of E.coli
fh	KSMBR1_1006-1007	fumarate hydratase
mdh	KSMBR1_3373	malate dehydrogenase
pckA	KSMBR1_0270	phosphoenolpyruvate carboxykinase
pyc	KSMBR1_1159	pyruvate carboxylase
oadA	KSMBR1_1800	oxaloacetate (OadA) or methylmalonyl-CoA decarboxylase (MmdA)
sucB	KSMBR1_3307-3308	2-oxoglutarate dehydrogenase complex E2 component
ppc	KSMBR1_1820	phosphoenolpyruvate carboxylase
**gluconeogensis**		
gpi	KSMBR1_2751	glucose-6-phosphate isomerase
fbp	KSMBR1_1273	fructose-1,6-bisphosphatase
fba_2	KSMBR1_1274	fructose-biphosphate aldolase
pfk	KSMBR1_2241	6-phosphofructokinase
tpi	KSMBR1_0356	triose-phosphate isomerase
gapN	KSMBR1_0641	NADP-dependent glyceraldehyde-3-phosphate dehydrogenase GapN
pgk	KSMBR1_1404	phosphoglycerate kinase
gpm	KSMBR1_0298	2,3-biphosphoglycerate-dependent phosphoglycerate mutase
apgM1	KSMBR1_1005	2,3-bisphosphoglycerate-independent phosphoglycerate mutase 1
eno	KSMBR1_1282	enolase
pyk	KSMBR1_0424	pyruvate kinase
ppsA	KSMBR1_2838	phosphoenolpyruvate synthase/ pyruvate phosphate dikinase
por	KSMBR1_2066-2069	pyruvate:ferredoxin-oxoacid:ferredoxin oxidoreductase
por	KSMBR1_3320-3321	pyruvate synthase
**pentose phosphate pathway**		
adh1a	KSMBR1_2588	alcohol dehydrogenase
zwf	KSMBR1_2015; KSMBR1_3962	glucose-6-phosphate dehydrogenase
pgl_1	KSMBR1_2014	6-phosphogluconolactonase
gnd1/2	KSMBR1_2016; KSMBR1_3963	gluconate-6-phosphate dehydrogenase, decarboxylating
tkt	KSMBR1_1753_KSMBR1_2299	transketolase
dxs_2	KSMBR1_2300	1-deoxy-D-xylulose 5-phosphate synthase (DXP synthase)
tktA	KSMBR1_3967	transketolase 1 thiamin-binding isozyme
fsa	KSMBR1_2523; KSMBR1_3948	fructose-6-phosphate aldolase 1
talAB	KSMBR1_2585; KSMBR1_3964	transaldolase
rpe	KSMBR1_1405	D-ribulose-5-phosphate 3-epimerase
rpiB	KSMBR1_0094; KSMBR1_1350	ribose-5-phosphate isomerase
**Wood-Ljungdahl pathway**		
codh/acsB	KSMBR1_1325	CO dehydrogenase/acetyl-CoA synthase alpha subunit
cdh/acsA	KSMBR1_1326	CO dehydrogenase/acetyl-coA synthase beta subunit (acsA)
fdhA_1	KSMBR1_1329	molybdopterin containing oxidoreductase (FdhA, nuoG, napA)
acsE	KSMBR1_1319	5-methyltetrahydrofolate/corrinoid Fe-S protein methyltransferase (acsE) of the CODH/ACS complex
acsD	KSMBR1_1320	small subunit of corrinoid FeS protein of the CODH/ACS complex (acsD)
acsF	KSMBR1_1321	nickel insertase (acsF) of CODH/ACS complex
metF	KSMBR1_2233	5,10-methylenetetrahydrofolate reductase
folD	KSMBR1_3289	bifunctional 5,10 methylene-tetrahydrofolate dehydrogenase/cyclohydrolase FolD
ftsH_6	KSMBR1_3178	formyltetrahydrofolate synthetase
fdhA_2	KSMBR1_3390	molybdopterin containing oxidoreductase (FdhA, nuoG, napA)
acsA	KSMBR1_2887	AMP-forming acetyl-CoA synthetase

Supplementary Table 1. List of central metabolism reactions and genes annotated in the *K. stuttgartiensis* genome (NCBI ID: LT934425.1).

Amino Acid	Mass (μmol/mgDW)	Std Dev (μmol/mgDW)
Ala	374.6	38.7
Arg	278.9	56.7
Asp	130.0	10.2
Glu	107.5	8.5
Gly	778.8	117.4
His	42.2	7.6
Ile	267.8	43.4
Leu	393.2	55.3
Lys	154.8	44.1
Met	218.9	58.4
Phe	205.4	43.0
Pro	460.0	96.1
Ser	436.5	94.1
Thr	381.0	86.5
Val	438.2	76.2

Supplementary Table 2. *K. stuttgartiensis* biomass amino acid composition.
